# Turkey-Syria Earthquake: The Importance of Providing a Direct Cross-border Support

**DOI:** 10.31662/jmaj.2023-0025

**Published:** 2023-06-12

**Authors:** Yudai Kaneda, Shiori Akashima

**Affiliations:** 1 School of Medicine, Hokkaido University, Hokkaido, Japan; 2 Department of Obstetrics and Gynecology, Shonan Kamakura General Hospital, Kanagawa, Japan

**Keywords:** Turkey-Syria Earthquake, International support, Support routes, Great East Japan Earthquake, Internal conflict

## Abstract

The 7.8 magnitude earthquake that occurred in southern Turkey on February 6, 2023, has resulted in over 40,000 and 5,000 confirmed deaths in Turkey and Syria, respectively, including substantial infrastructure damage. While Turkey has received assistance from various countries and international organizations, the level of support Syria has received remains unclear. The Assad regime has demanded that aid be sent to the capital Damascus, but this may hinder the delivery of assistance to the areas most severely affected by the earthquake because the affected regions were mainly controlled by rebel forces.

One of the biggest challenges in providing aid is accessing the affected region, as the earthquake occurred close to the border between Syria and Turkey, where roads and other infrastructure are poorly constructed. Furthermore, the northwest area of Syria shelters many internally displaced people, and more than 50% of medical facilities are not functioning due to the ongoing conflict, making the situation more hazardous.

In light of the experience of the Great East Japan Earthquake, securing direct support routes and dispatching medical personnel to the affected areas is crucial for a gradual recovery from the disaster. Therefore, it is essential for the international community, including Japan, to negotiate with the Assad regime to expand direct support routes and provide support for the dispatch of medical personnel who will remain and work in the affected areas. Humanitarian assistance and political issues should be kept separate to avoid further hindrances to aid delivery.

The need for international cooperation, regardless of the underlying political issues, to save the local people from disasters in countries experiencing internal conflicts is currently being emphasized ^(1)^. In this context, the magnitude 7.8 Turkey-Syria earthquake occurred on February 6, 2023. More than 40,000 people in Turkey and 5,000 in Syria have been confirmed dead. Although Turkey has received assistance from 70 countries, such as the United States, the United Kingdom, and Russia, including 14 international organizations, the situation of support for Syria from the international community is unclear.

This is mainly due to the internal conflict within Syria that began in 2011, which has divided the country into several ruling groups and resulted in a lack of domestic coordination. The Assad regime has been internationally criticized and isolated for its thorough suppression of rebel forces and is under severe sanctions. Consequently, the current report revealed that more than 80% of the Syrian people live in poverty, less than US$2 per day and more than 9 million people live in food scarcity ^(2)^. Furthermore, in Syria, more than 50% of medical facilities are reportedly dysfunctional due to the prolonged internal conflict ^(3)^, and the Syrian people are desperate to recover from the disaster on their own. As for international assistance for the earthquake, the Assad regime has demanded that all aid be sent to Damascus’s capital, including aid to areas outside their control. So far, Iran, Egypt, India, and some other countries have sent aid supplies directly to airports under the Assad regime’s control.

However, some areas most severely affected by the earthquake were regions controlled by rebel forces. Although international aid should be provided based on formal requests from the affected country’s government to protect its sovereignty, delivering aid to the Assad regime by air may prevent timely assistance to victims in rebel-controlled areas, making it increasingly important to provide direct local assistance from abroad. Therefore, access to the area for humanitarian activities has become one of the major challenges since the earthquake occurred near the border between Syria and Turkey, where roads and other infrastructure are not well-developed. Indeed, before the earthquake, only one road in this area could carry supplies directly from Turkey to Syria, as rebel forces controlled this area. This is also an area where many internally displaced people, mainly women and children, have taken shelter from the internal conflict ^(3)^, thus making the situation much more hazardous.

Considering the experience of the Great East Japan Earthquake, one of the most critical initial responses was to secure 16 emergency routes to the affected areas by removing obstacles and opening access roads, known as the "teeth of a comb strategy," resulting in 97% of them being passable within one week ^(4)^. This enabled the direct provision of necessary support and the dispatch of medical professionals from other prefectures, which considerably contributed to promptly improving the health of the affected areas. Despite different backgrounds, it is essential to recover from the disaster to prepare support routes as quickly as possible and to dispatch researchers and medical personnel to the affected areas so that the medical system can be set up promptly ^(4)^.

In this context, the continued failure to secure support routes to Syria could lead to more deaths and injuries in the affected areas. Despite the differences in social stability, political, and cultural backgrounds between the current earthquake and the Great East Japan Earthquake, regardless of the political issues, it is crucial for the international community, including Japan, to negotiate with the Assad regime to expand direct support routes and to assist dispatching the medical personnel who will remain and work in the affected areas. Additionally, the purposes of the World Health Organization Emergency Medical Team Initiative include simultaneously enhancing the timeliness and quality of medical services through collaboration among domestic and international emergency medical teams, promoting rapid response capabilities in the immediate aftermath of disasters, outbreaks, and other emergencies, and strengthening the capacity of national health systems to lead coordination efforts ^(5)^. Thus, considering that the fundamental cause of the difficulty in providing international aid to Syria stems from the ongoing civil war, which has made it challenging to trust the current regime, it is crucial to undertake efforts to dispel tensions between the Assad regime and the international community by continuously implementing peacekeeping activities and long-term support, rather than solely focusing on short-term assistance in the aftermath of an earthquake. We look forward to establishing an international cooperative framework between Turkey and Syria and hope for a rapid reconstruction of both countries.

## Article Information

### Conflicts of Interest

None

### Author Contributions

Conception, designing of the study, and writing this paper; Kaneda Y

Critical revision of the paper; Akashima S

All authors have read the final draft and approved the submission.
